# Effects of HDAC inhibitors on neuroblastoma SH-SY5Y cell differentiation into mature neurons via the Wnt signaling pathway

**DOI:** 10.1186/s12868-023-00798-0

**Published:** 2023-05-01

**Authors:** Jiyun Choi, Jinsu Hwang, Mahesh Ramalingam, Han-Seong Jeong, Sujeong Jang

**Affiliations:** Department of Physiology, Chonnam National University Medical School, Hwasun-gun, Jellanamdo 58128 Republic of Korea

**Keywords:** HDAC inhibitor, MS275, VPA, Neuronal differentiation, Wnt signaling pathway

## Abstract

Histone deacetylase (HDAC) inhibitors affect cell homeostasis, gene expression, and cell cycle progression and promote cell terminal differentiation or apoptosis. However, the effect of HDAC inhibition on SH-SY5Y cells, which are neuroblastoma cells capable of differentiating into neurons under specific conditions, such as in the presence of retinoic acid (RA), is unknown. In this study, we hypothesized that HDAC inhibitors induced the neuronal differentiation of SH-SY5Y cells. To test this hypothesis, we used phase contrast microscopy, immunocytochemistry (ICC), qPCR, and western blotting analysis. MS-275 and valproic acid (VPA), two HDAC inhibitors, were selected to evaluate neuronal differentiation. It was confirmed that cells treated with MS-275 or VPA differentiated into mature neurons, which were distinguished by bipolar or multipolar morphologies with elongated branches. In addition, the mRNA expression of neuronal markers (*Tuj1* and *NEFH*) and the oligodendrocyte marker (*CNP*) was significantly increased with MS-275 or VPA treatment compared to that with RA treatment. In addition, the protein expression of the other neuronal markers, Tuj1 and NeuN, was highly increased with HDAC inhibitor treatments compared to that with RA treatment. Furthermore, we confirmed that noncanonical Wnt signaling was upregulated by HDAC inhibitors via MAPK signaling and the Wnt/JNK pathway. Therefore, both MS-275 and VPA promoted the differentiation of SH-SY5Y cells into mature neurons via the Wnt signaling pathway.

**Supplementary Information:**

The online version contains supplementary material available at 10.1186/s12868-023-00798-0.

## Introduction

Histone deacetylase (HDAC) inhibitors affect various cellular signals, such as cell proliferation, terminal differentiation, apoptosis, cell homeostasis, cell cycle arrest, and gene expression [1, 2]. When histone acetyltransferase (HAT) transfers the acetyl groups of acetyl-CoA to the lysine residues in the NH_2_ terminal tails of core histones, the chromosome is uncoiled into chromatin, making transcription easier [3]. When HDACs remove the acetyl group, the chromatin condenses back into a chromosome [1, 4]. This epigenetic mechanism activates cell proliferation and makes transcription difficult [1]. Conversely, HDAC inhibitors prevent histone lysine residues from being deacetylated. Because acetylation leads to uncoiled chromatin, it activates transcription and affects gene expression.

MS-275, which is an HDAC1, HDAC2, and HDAC3 inhibitor, is a candidate for treating autism and cancers such as advanced breast cancer and metastatic lung cancer [1, 5, 6]. Some studies suggest that MS-275 improves social and synaptic function associated with autism and alleviates postoperative cognitive dysfunction by reducing hippocampal neuroinflammation [6, 7]. MS-275 also induces the odontoblast differentiation of human dental pulp stem cells and the neurogenic differentiation of human adipose tissue-derived mesenchymal stem cells [1, 8]. Valproic acid (VPA) is a class I and class IIa HDAC inhibitor that has been clinically used as a therapeutic agent for the treatment of breast cancer, intracranial glioma, bipolar disorder, brain metastases, spinal muscular atrophy, and migraine [1, 9, 10]. VPA can act on the cardiovascular system and can be used to manage diabetes mellitus; it also has anti-inflammatory and neuroprotective effects [11]. VPA has been shown to alter estrogen receptor status and metastatic potential and modulates the tumor immune response of breast cancer cells [9]. Other studies suggest that VPA promotes the neuronal differentiation of adipose tissue-derived stem cells via the Wnt signaling pathway or the inducible nitric oxide synthase-soluble guanylyl cyclase signaling pathway [1, 12]. One study also showed that VPA enhances the neuronal differentiation of spiral ganglion neural stem cells with much longer neurite outgrowth via the Wnt/β-catenin signaling pathway [13].

The Wnt signaling pathway, which is associated with proliferation, apoptosis, differentiation, and the cell cycle, can be classified into the canonical and noncanonical pathways [14]. In the canonical pathway, when Wnt (Wnt1, Wnt2, or Wnt3α) is activated, it can bind with the frizzled (Fzd) receptor and stimulate the coreceptors, low-density lipoprotein receptor-related protein 5 (LRP5) or LRP6 and dishevelled (Dvl), to inhibit β-catenin proteolysis [15]. β-Catenin dissociates from the destruction complex, composed of Axin1, adenomatosis polyposis coli (APC), phosphorylated glycogen synthase kinase 3β (p-GSK3β), and casein kinase 1 (CK1), and translocates into the nucleus leading to gene transcription [15, 16]. The noncanonical pathway, which does not regulate β-catenin, has the following two downstream signals; the Wnt/Ca^2+^ pathway and the Wnt/JNK pathway. Wnt4, Wnt5α, or Wnt11, which are known to be noncanonical forms of Wnt, bind with Fzd and Dvl, which leads to phospholipase C (PLC) activation in the Wnt/Ca^2+^ pathway. Increased Ca^2+^ stimulates protein kinase C (PKC), calcineurin, and calcium/calmodulin-dependent protein kinase II (CaMKII), and nuclear factor of activated T-cell (NFAT) translocates into the nucleus leading to gene transcription [14]. Several studies have demonstrated that activated Dvl stimulates phosphorylated ERK and JNK and leads to gene transcription and neuronal differentiation via c-Jun in the Wnt/JNK pathway [1, 14].

Although some studies have assessed neuronal differentiation using HDAC inhibitors, the effects of neuronal differentiation on SH-SY5Y cells by HDAC inhibitors have not been well demonstrated [17–19]. Therefore, in this study, we confirmed the effect of HDAC inhibitors on the differentiation of SH-SY5Y cells into mature neurons via the Wnt signaling pathway.

## Materials and methods

### Cell lines and reagents

We used the human neuroblastoma cell line, SH-SY5Y (RRID: CVCL_0019l ATCC® CRL-2266), which can differentiate into mature neuronal cells under certain conditions. The cells were grown in Dulbecco's modified Eagle's medium (DMEM; WELGENE, Gyeongsan-si, Republic of Korea) supplemented with 10% fetal bovine serum (FBS; EMD Millipore Corp., Burlington, Massachusetts, USA), 1% penicillin–streptomycin (WELGENE), and 0.2% amphotericin B (Life Technologies Corporation, Carlsbad, USA) at 37℃ in a 5% CO_2_ incubator. Retinoic acid (RA; 10 μM), a known reagent for the neuronal differentiation of SH-SY5Y cells, was dissolved in dimethyl sulfoxide (DMSO; Sigma–Aldrich, Burlington, MA, USA) and used as a positive control in this study. We selected two HDAC inhibitors, MS-275 and VPA, and dissolved them in DMSO as described in a previous study [1]. The concentration of the HDAC inhibitors was determined according to several references based on the inhibition of HDAC, activation of HAT [20–25], and neuronal differentiation [1, 26–28]. Differences in shape were confirmed by taking images using a phase-contrast microscope; neuronal differentiation occurred most actively with HDAC inhibitor treatment. In addition, the cell viability was determined by MTT assay and then the concentration of the HDAC inhibitors was chosen.

### Immunocytochemistry (ICC)

For ICC, the cells (5 × 10^4^ cells/mL) were cultured on poly-L-lysine-coated aclar plastic coverslips as described previously [29]. Medium containing 1% FBS (EMD Millipore Corp.) was changed to medium containing the HDAC inhibitors. The cells were fixed with 4% paraformaldehyde (PFA; T&I Co., Chuncheon-si, Republic of Korea) for 15 min at room temperature. The cells were then blocked with 0.5% Triton X-100 (Sigma–Aldrich) for 20 min and 10% normal goat serum (NGS; Vector Laboratories, Inc., Burlingame, USA) for 30 min. Primary antibodies were added for 1.5 h, and the secondary antibodies were then added and kept in the dark for 1 h. For staining nuclei, 4′,6-diamidino-2-phenylindole (DAPI,  1 μg/mL, Life Technologies Corporation) was added for 30 min. The cells were then imaged with a Zeiss LSM510 confocal microscope (Carl Zeiss, Jena, Germany) after mounting [29]. The primary antibodies used were microtubule-associated protein 2 (MAP2, 1:200; Cell Signaling Technology, Danvers, USA) and neurofilament-H (NFH, 1:400; Cell Signaling Technology). Alexa 488-conjugated goat anti-rabbit (Invitrogen Co., Waltham, USA) and Alexa 594-conjugated goat anti-mouse (Invitrogen Co.) were used as the secondary antibodies. All experiments were repeated at least three times.

### RNA isolation and cDNA synthesis

For qPCR, cells (5 × 10^5^ cells/mL) treated with HDAC inhibitors were harvested using TRIzol (TaKaRa Co., Shimogyo-ku, Japan) as described previously [1]. Recombinant RNase Inhibitor (TaKaRa Co.), Go script Buffer Mix Oligo dT (Promega Co., Madison, USA), and Go script Enzyme Mix (Promega Co.) were added to synthesize cDNA according to the manufacturer’s instructions. A PCR instrument (Takara Co. ) was used for denaturing RNA and synthesizing cDNA. The synthesized cDNA was stored at 4 °C.

### Quantitative PCR (qPCR)

qPCR analyses were performed with SYBR Green Premix Ex Taq (Takara Co. ) and LightCycler 480 II (Roche Holding AG., Basel, Swiss) at 60 °C, as recommended by the manufacturer’s instructions. All primers listed in Table 1 were purchased from Bioneer and CosmoGenetech (Seoul, Korea). Each sample was analyzed in three replicate reactions of 10 μL.

**Table 1:** Sequence of qPCR primers

Gene	Forward (5′-3′)	Reverse (5′-3′)	References
*MAP2*	CGGATCAACAGACAACATC	CTGTGGCGGATGTTCTTC	NM_001375534.1 [30]
*NEFL*	AGCCGTACTACTCGACCTCC	GACTGGGCATCAACGATCCA	NM_006158.5 [31]
*NEFH*	GTGGACCTGCAGAAGAAG	CACCTCTTCCTGGTGGTG	NM_021076.4 [32]
*NEFM*	GCGCAAAGACTACCTGAAG	GGCCTGGTGCATATTCTG	NM_005382.2 [33]
*CNP*	CAAGATGTCATCCTCAGGG	GAGCGTCTTGCACTCTAG	NM_001330216.2 [34]
*Tuj1*	CGATGCCAAGAACATGATG	CTCATCGACCTCCTTCATG	AF 141349.1 [35]
*GFAP*	GGGCAGAGATGATGGAGC	CCTTGTTTTGCTGTTCCAG	NM_002055.5 [31]
*GAPDH*	GACAGTCAGCCGCATCTTCT	GCGCCCAATACGACCAAATC	NM_002046.7 [36]

*MAP2* Microtubule-associated protein 2; *NEFL* Neurofilament light chain; *NEFH* Neurofilament heavy chain; *NEFM* Neurofilament medium chain; *CNP* 2′3′-Cyclic-nucelotide 3′-phosphodiesterase; *Tuj1* β3-Tubulin; *GFAP* Glial fibrillary acidic protein; *GAPDH* Glyceraldehyde 3-phosphate dehydrogenase

### Western blotting analysis

For western blotting analysis, cells (5 × 10^5^ cells/mL) were cultured and lysed in a lysis buffer (1 M Tris pH 7.5, 1 M NaCl, 0.5 M EDTA, 10% Nonidet P-40, 100% glycerol, 50 mg/mL leupeptin, 50 mg/mL aprotinin, 0.2 M PMSF, 0.1 M Na_3_VO_4_, 1 M NaF) as described previously [37]. The western blot membranes containing proteins were incubated with specific antibodies against neurofilament-heavy chain (NFH, 1:1000), Tuj1 (1:1000), synaptophysin (SYP, 1:1000), neuronal nuclei (NeuN, 1:2000), Wnt5α/β (1:1000), Fzd5 (1:1000), Dvl2 (1:1000), Dvl3 (1:1000), Axin1 (1:1000), PKC (1:1000) p-ERK (1:1000), ERK (1:1000), p-JNK (1:1000), JNK (1:1000), c-Jun (1:1000), and GAPDH (1:3000) [37, 38]. Horseradish peroxidase (HRP)-conjugated goat anti-rabbit IgG antibody and HRP-conjugated goat anti-mouse IgG antibody were used as the secondary antibodies. All primary and secondary antibodies were purchased from Cell Signaling Technology and Santa Cruz Biotechnology. The immunoblotted bands were measured by Immobilon Crescendo Western HRP substrate (EMD Millipore Corporation). ImageJ software was used for quantitative analysis of immunoblotted bands [37]. All experiments were repeated at least three times.

### Statistical analysis

Statistical analyses were performed with GraphPad Prism® 5.0 software (GraphPad Software Inc., San Diego, CA, USA). Differences among groups were assessed using two-way ANOVA. Repeated measures were assessed by ANOVA with significance levels of *p < 0.05, **p < 0.01, and ***p < 0.001 compared to the control; and ^#^p < 0.05, ^##^p < 0.01, and ^###^p < 0.001 compared to the RA-treated group. If mean values between populations showed statistically significant changes, they were analyzed by post–hoc tests (Bonferroni). ICC, qPCR, and western blotting data were analyzed using one–way ANOVA and post–hoc tests (Tukey’s).

## Results

### Morphological changes

**Fig. 1:**
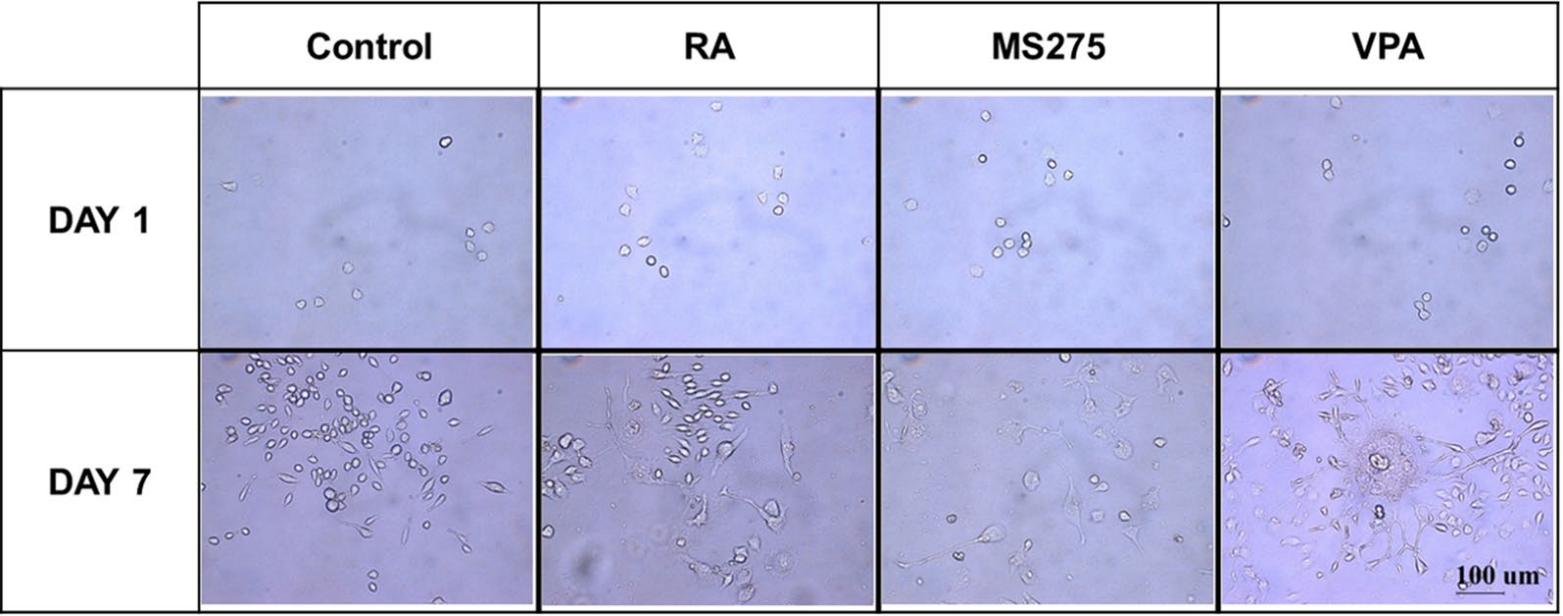
Neuron-like morphology after HDAC inhibitor treatment. SH-SY5Y cells were cultured with RA (10 μM), MS-275 (500 nM), or VPA (20 μM) and images were taken on the 1st and 7th days under a microscope. HDAC inhibitors (MS-275 and VPA) induced neuronal differentiation effectively compared to control and RA treatments. The scale bar represents 100 μm

To assess whether the cell morphology was changed with HDAC inhibitor treatment, we imaged cells treated with HDAC inhibitors under a microscope for 1 week. After RA and HDAC inhibitor (MS-275 or VPA) treatment, the cells had bipolar or multipolar neurites with elongated branches compared to the control cells. These morphological changes suggest that the HDAC inhibitors induced the differentiation of SH-SY5Y cells into mature neurons (Fig. 1).

We measured cell death in different concentrations following treatment with HDAC inhibitors, and the chosen concentrations did not have an effect on the death of the cells (Additional file 1: Fig. S1). These data confirmed that the cell viabilities were decreased with high concentrations of HDAC inhibitors [21, 25, 39]. In addition, to investigate the inhibition of histone deacetylation with HDAC inhibitor treatment, the protein expression of HDAC was determined using western blotting (Additional file 1: Figs. S2 and S3). We figured out that the concentration of HDAC inhibitors affected histone modification, as in previous reports [20–25].

### Neuronal differentiation of SH-SY5Y cells with HDAC inhibitors

**Fig. 2:**
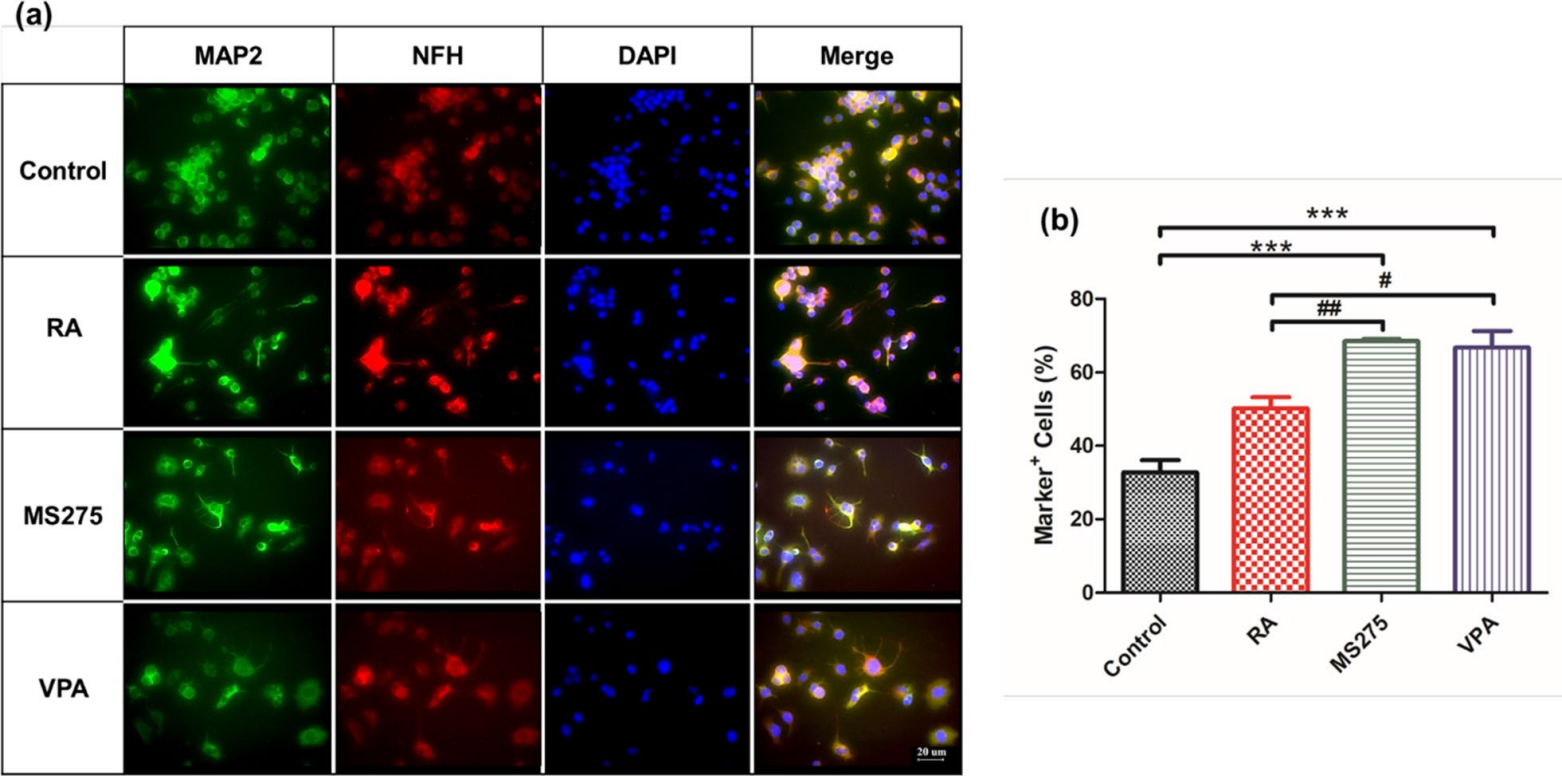
Analysis of neuronal markers by fluorescent immunocytochemistry. **a** Fluorescent immunocytochemistry showing MAP2-positive cells (green) and NFH-positive cells (red) following HDAC inhibitor treatment compared to the control and RA treated cells. DAPI (blue) was used to stain the nuclei of cells. The scale bar represents 20 μm. **b** The number of positive cells that expressed MAP2 and NFH was measured. The ratio of positive cells to nuclei was calculated for each group (n = 4). All experiments were repeated at least three times. ***p < 0.001 compared to the control group, ^#^p < 0.05 and ^##^p < 0.01 compared to the RA group

To determine the increase in neuronal markers, we analyzed SH-SY5Y cells treated with HDAC inhibitors using immunocytochemistry with primary antibodies against MAP2 and NFH. MAP2 is located in the dendrites of neurons and the soma, and NFH, a component of the cytoskeleton, is usually located in axons. Figure 2 shows the neurite outgrowth and morphology of mature neurons treated HDAC inhibitors compared to the control and RA groups. Neuronal marker-positive cells were increased with the following HDAC inhibitor treatments; 32.79 ± 3.313% in control, 50.18 ± 3.037% in RA, 68.48 ± 0.5366% in MS-275, and 66.74 ± 4.477%, in VPA (Fig. 2b).

### Neuronal marker genes were highly expressed with HDAC inhibitor treatments

**Fig. 3:**
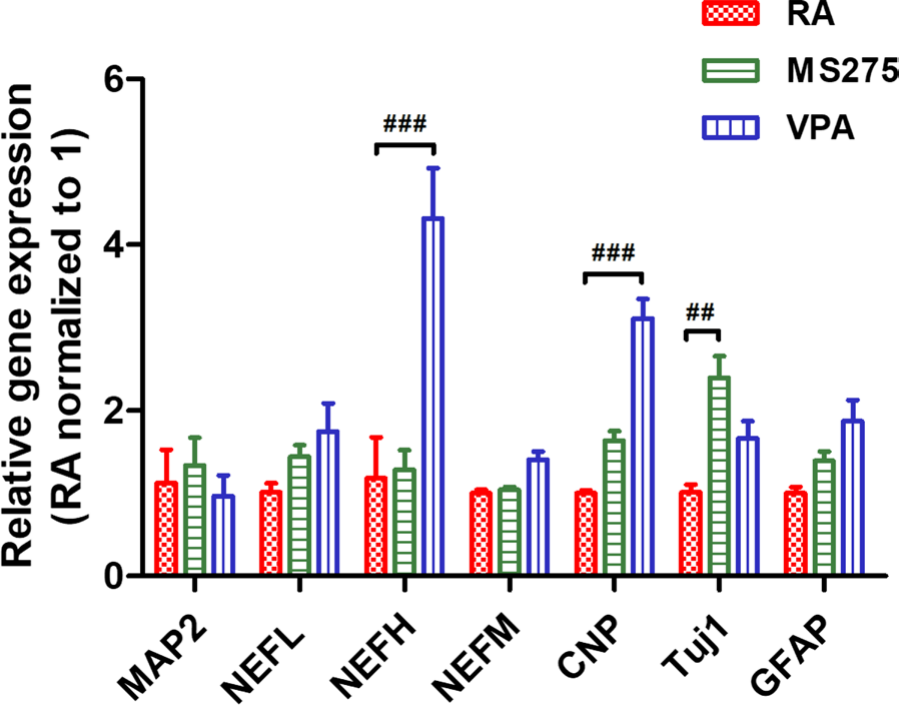
qPCR assay. We analyzed the gene expression of *MAP2*, *NEFL*, *NEFH*, *NEFM*, *CNP*, *Tuj1*, and *GFAP* by qPCR. The gene expression of most neuronal markers was increased with HDAC inhibitor treatment compared to RA treatment. The mRNA expression was normalized to the RA-treated cells. *GAPDH* was used as a control. All experiments were repeated at least three times. ^##^p < 0.01 and ^###^p < 0.001 compared to the RA group

In a previous study, we investigated the effect of HDAC inhibitors on stem cell differentiation into mature neurons, oligodendrocytes, or astrocytes [1]. Here, we determined that the mRNA expression of *NEFH*, a mature neuronal marker, and *CNP*, an oligodendrocyte marker, was significantly increased with VPA group compared to RA group (Fig. 3) using qPCR analyses. Interestingly, the expression of *Tuj1* was increased with MS-275 treatment. These results suggest that MS-275 and VPA are effective inducers of neuronal differentiation and are, therefore, novel drugs for SH-SY5Y cell differentiation [40, 41].

### Regulation of neuronal differentiation via Wnt/JNK signaling

**Fig. 4:**
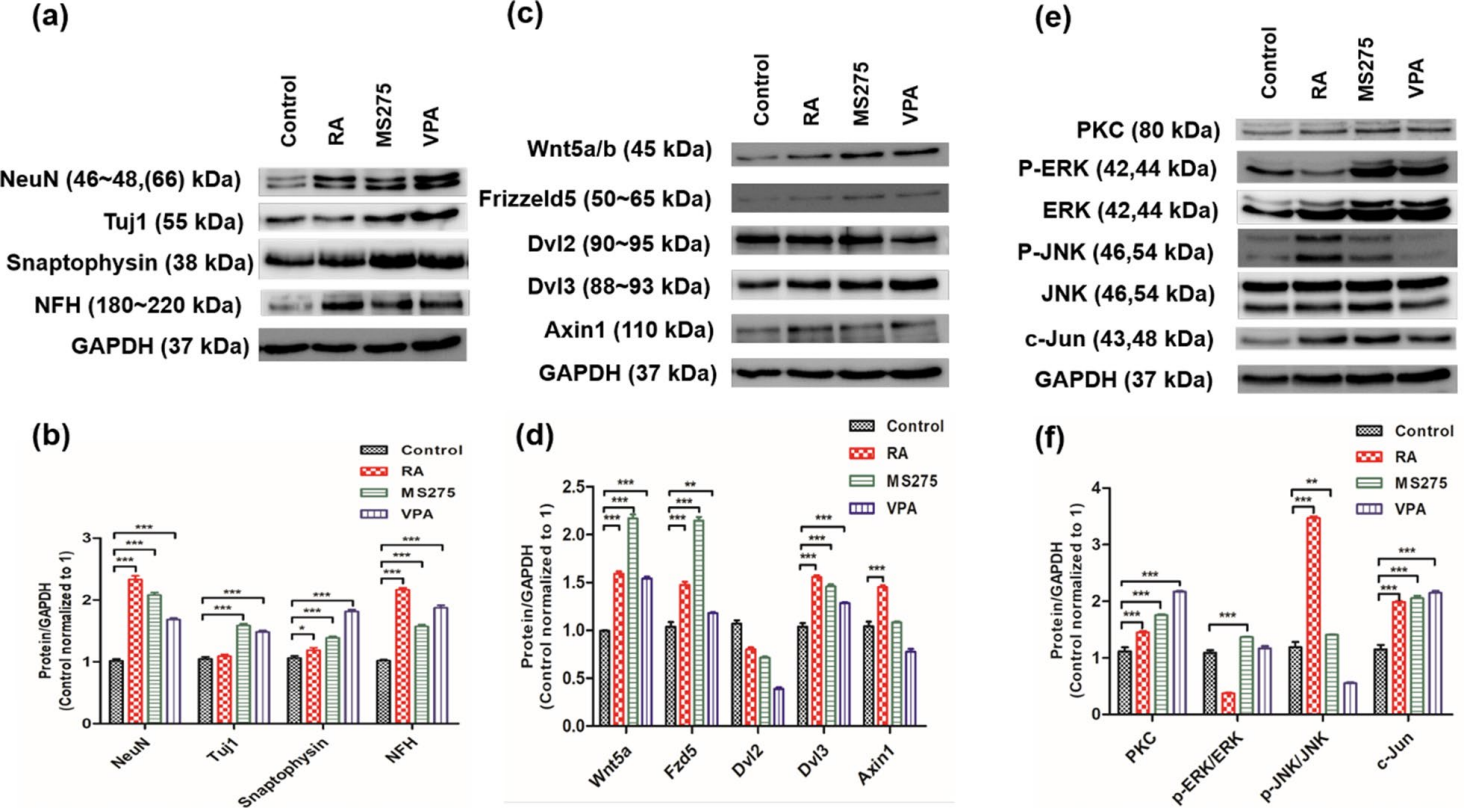
Expression of neuronal markers and Wnt-related signals. The levels of neuronal markers (**a**, **b**), Wnt-related proteins (**c** and **d**), and MAPK signaling (**e** and **f**) were determined via western blotting analysis. GAPDH was used as a control. Original uncut western blotting images are shown in Additional file 1: Figs. S4, S5, S6. All experiments were repeated at least three times. *p < 0.05, **p < 0.01, and ***p < 0.001 compared to the control group

We studied the protein expression of neuronal markers, such as NFH, Tuj1, SYP, and NeuN, by western blotting analysis. The expression of NeuN, Tuj1, SYP, and NFH was increased with HDAC inhibitors and RA treatment compared to the control group. Interestingly, Tuj1 and SYP were significantly upregulated following treatment with HDAC inhibitors compared that with to RA treatment (Fig. 4a, b, and Additional file 1: Fig. S4).

Previously, our lab and several others studied the Wnt signaling pathway, which is related to neuronal differentiation [1, 38, 42, 43]. Therefore, here we investigated whether the Wnt signaling pathway affects neuronal differentiation following HDAC inhibition. Interestingly, Wnt5α/β, which is related to noncanonical Wnt signaling, was highly increased with the HDAC inhibitors and RA treatment compared to the control group. Similarly, Fzd5, which is located around the cytoplasmic membrane and binds to canonical and noncanonical Wnt, was increased with the HDAC inhibitor and RA treatment compared to the control group. Dvl is connected with Fzd and regulates downstream signaling. Dvl2 was decreased while Dvl3 was increased following treatment with the HDAC inhibitors. Importantly, Axin1, which interacts with GSK3β and β-catenin, was decreased with VPA treatment compared to the control group (Fig. 4c, d, and Additional file 1: Fig. S5).

**Fig. 5:**
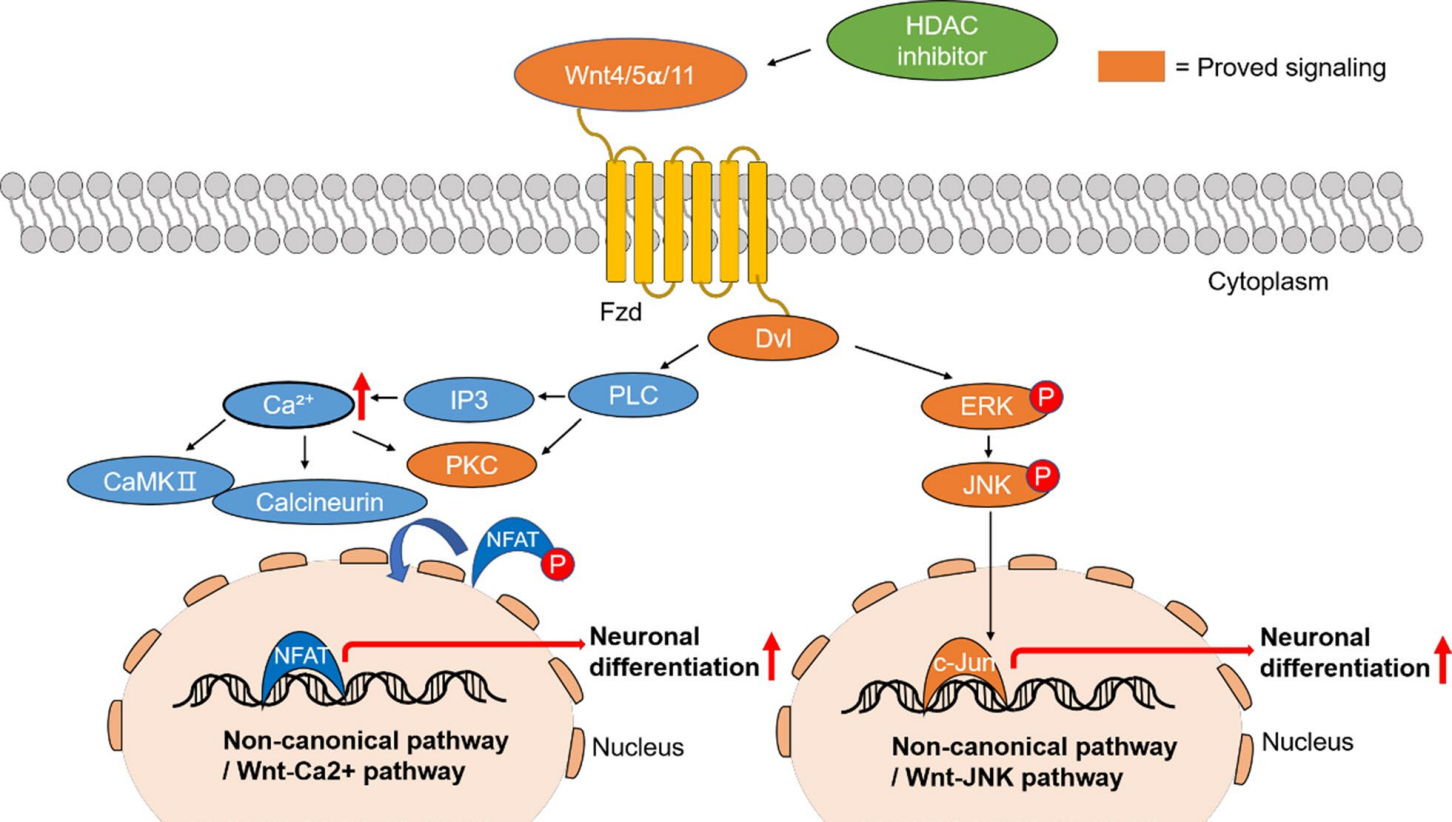
Noncanonical Wnt signaling for neuronal differentiation with HDAC inhibitors. HDAC inhibitors activate Wnt4, Wnt5α, Wnt 11, and the Fzd receptor. Fzd activates Dvl and stimulates the Wnt/Ca^2+^ or Wnt/JNK pathway. In the Wnt/Ca^2+^ pathway, activated Dvl promotes Ca^2+^, which stimulates PKC. In the Wnt/JNK pathway, activated Dvl promotes phosphorylated ERK and phosphorylated JNK. Finally, c-Jun, a transcription factor, binds to DNA and promotes neuronal differentiation. Here, we demonstrate that Wnt4/5α/11 can modulate Dvl/PKC signaling and the Dvl/p-ERK/p-JNK pathway for neuronal differentiation following HDAC inhibition. Orange color indicates the signaling observed in this study

The noncanonical Wnt signaling pathway independent of β-catenin is classified as the Wnt/Ca^2+^ and Wnt/JNK pathways. To assess the activation of noncanonical Wnt signaling with HDAC inhibitor treatment, phosphorylation-specific antibodies were used as previously described [1]. In the Wnt/Ca^2+^ pathway, the expression of PKC was stimulated by HDAC inhibitor treatment. In the Wnt/JNK pathway, phosphorylated ERK was activated by HDAC inhibitor treatment. Interestingly, the expression of phosphorylated JNK was highly upregulated with RA treatment. The expression of c-Jun, the transcription factor of the noncanonical pathway, was increased with MS-275, VPA, and RA treatments compared to the control (Fig. 4e, f, and Additional file 1: Fig. S6). Taken together, these data show that HDAC inhibitors induced neuronal differentiation of SH-SY5Y cells by activating the noncanonical Wnt signaling pathway, specifically, the Wnt/JNK pathway (Fig. 5).

## Discussion

HDAC inhibitors are related to the induction of apoptosis and autophagy, cell cycle arrest, hormone signaling, immune effects, neuroprotection, and neuronal differentiation [1, 2, 44, 45]. Some HDAC inhibitors, such as romidepsin, vorinostat, or ricolinostat, are already approved or advanced as clinical candidates for cancer therapy [46]. MS-275, another HDAC inhibitor, decreases cell proliferation and induces the differentiation of human dental pulp stem cells into odontoblast-like cells [8] and human mesenchymal stem cells [1]. MS275 also has been known to differentiate U87MG glioblastoma multiforme cells into neural cells when it synergized with 8-CPT-cAMP [47]. Another HDAC inhibitor, VPA, has been known to effective inducer of differentiation. For example, it could stimulate neurite outgrowth and prolong cell survival via the ERK [45] and JNK [1] pathway. In addition, VPA could induce the differentiation of PC12 cells which are the neuroblastoma cell into mature neurons [48]. Other groups were reported that adipose tissue-derived stem cells could also differentiate into neuronal cells by treating VPA [12, 49]. Similarly, it has been reported that VPA has a potential as an agent of neurogenic differentiation via activating histone H3 core acetylation in embryonic stem cells [50] and osteogenic differentiation in tonsil-derived mesenchymal stem cells [51]. Animal studies have also shown that VPA treatment in the rat cerebral cortex induced differentiation via activation of the ERK-P21 Cip/WAFI pathway [45, 52]. In this study, we demonstrated that MS-275 and VPA were effective inducers of neuronal differentiation via the activated ERK/JNK pathway by Wnt signaling.

We investigated the effect of HDAC inhibitors (MS-275 and VPA) on neuronal differentiation by observing morphology via phase contrast and ICC. The expression of both MAP2 [53] and NFH [54, 55] was markedly increased with HDAC inhibitors (MS-275 or VPA) compared to the control and RA-treated cells. Previous studies have reported that HDAC inhibitors can regulate cell proliferation and cell apoptosis in numerous cancer cell lines [56, 57]. Similarly, cell proliferation was not increased with MS275 and VPA treatment. However, the neurites were elongated and the numbers increased (Figs. 1 and 2).

HAT is important for astrocyte differentiation, and HDAC is important for oligodendrocyte differentiation [1, 58]. We also obtained the similar results by qPCR analysis that acetylation via HDAC inhibitors promotes the differentiation of oligodendrocytes. In the western blotting analysis, we assessed whether the protein expression of NeuN, Tuj1, SYP, and NFH was increased with HDAC inhibitors. NeuN, which is a soluble nuclear protein related to terminal neuronal differentiation, was used to estimate neuronal cell loss in NDDs [55]. Tuj1 can be used to determine microtubule protofilament stability and was used to evaluate the expression of neuronal markers [59]. SYP, which is a membrane glycoprotein located in presynaptic vesicles, regulates the endocytosis of synaptic vesicles [55, 60]. NFH plays a role in axonal architecture and neurite outgrowth [55]. In this study, the expression of NeuN, Tuj1, SYP, and NFH was highly increased with HDAC inhibitors compared to that with the control. In addition, the levels of Tuj1 and SYP were enhanced with HDAC inhibitors compared to that following RA treatment. These data suggest that MS-275 and VPA could be used as effective inducers of neuronal differentiation (Figs. 3, 4 and Additional file 1: Fig. S4 ).

The Wnt signaling pathway is classified as either the canonical pathway, which is dependent on β-catenin, or the noncanonical pathway, which is independent of β-catenin [15, 16]. Noncanonical Wnt signaling is further classified as the Wnt/Ca^2+^ and Wnt/JNK pathways. In our results, Wnt4, Wnt5α, and Wnt11 were activated by the HDAC inhibitors and bound to the Fzd receptor to stimulate Dvl (Fig. 4 and Additional file 1: Figs. S5, S6). Stimulated Dvl activates PLC and PKC, and the subsequent signals increase Ca^2+^ via IP3-activated PKC, calcineurin, and CaMKII [14]. Activated calcineurin removes the phosphate group from NFAT, and NFAT translocate to the nucleus to activate transcription and differentiation in the Wnt/Ca^2+^ pathway [14]. In the Wnt/JNK pathway, activated Dvl stimulates phosphorylated ERK and JNK and activates c-Jun, a transcription factor, to increase transcription and cell differentiation. It has been reported that a single Wnt5α can promote neuronal differentiation via the Wnt5α/JNK pathway in human adipose tissue-derived stem cells [38]. Another study has reported that VPA can induce the differentiation of human pluripotent stem cells into spermatogonial stem-cell like cells by activating Wnt signaling pathway [61]. We obtained similar results in SH-SY5Y cells treated with HDAC inhibitors. The increased expression of Wnt5α, Fzd5, PKC, p-ERK, p-JNK, and c-Jun with HDAC inhibitors suggests that MS-275 and VPA activated the noncanonical Wnt signaling pathway to promote the neuronal differentiation of SH-SY5Y cells. In addition, MS-275 induces the PP2A B-type subunit PR130, which is a modulator of Wnt signaling, in various cell types [62–64]. The PP2A-PR130 complex modulates the Wnt signal transduction pathway by restricting the functions of the intracellular Wnt regulator naked cuticle [62]. The expression of PR130 might be increased with HDAC inhibitor treatment in NB cells. This assumption requires further experimental evidence.

Some animal studies have suggested that HDAC inhibitors can be used as potential future therapeutics for neurological diseases. For example, it has been known that VPA promotes hippocampal neurogenesis and cell proliferation via the Wnt/β-catenin signaling pathway in the transgenic mouse model of Alzheimer’s diseases [65], Parkinson’s disease [66], and amyotrophic lateral sclerosis [66, 67]. In addition, VPA has been reported to induce synaptogenesis, axonal regeneration, and synaptic plasticity in the mice model of traumatic brain injury [68]. Several studies have been reported that Wnt regulators have been used as potential drugs for cancer treatment [1, 16, 69]. It has also been reported that the Wnt signaling pathway could be a therapeutic target to induce bone growth and skeletal tissue regeneration [70]. So, we expect that the Wnt signaling pathway could also be used as a therapeutic target for neurological disorders such as Alzheimer’s disease. Our next goal is to find a novel drug targeting the Wnt signaling pathway for neurological disorders and to investigate neuronal differentiation by histone acetylation in other cell lines. Taken together, these results indicate that MS-275 and VPA promote transcription and neuronal differentiation in SH-SY5Y cells by acetylation of lysine residues mediated by the noncanonical Wnt signaling.

## Supplementary Information

**Additional file 1**. Additional figures.

## Data Availability

The datasets generated or analyzed during this study are available in  the article and Additional file.

## Abbreviations

HDAC: Histone deacetylase
NB: Neuroblastoma
RA: Retinoic acid
VPA: Valproic acid
ICC: Immunocytochemistry
qPCR: Quantitative PCR
MAP2: Microtubule-associated protein 2
NEFL: Neurofilament light chain
NEFM: Neurofilament medium chain
NEFH: Neurofilament heavy chain
Tuj1: β-Tubulin
CNP: 2',3'-Cyclic-nucleotide 3'-phosphodiesterase
GFAP: Glial fibrillary acidic protein
GAPDH: Glyceraldehyde 3-phosphate dehydrogenase
NeuN: Neuronal nuclear protein
SYP: Synaptophysin
Fzd: Frizzled
Dvl: Dishevelled
PKC: Protein kinase C
ERK: Extracellular signal-regulated kinase
JNK: C-Jun N-terminal kinase
APC: Adenomatosis polyposis coli
p-GSK3β: Phosphorylated glycogen synthase kinase 3β
CK1: Kinases casein kinase 1
PLC: Phospholipase C
NFAT: Nuclear factor of activated T-cell
DMEM: Dulbecco's modified Eagle's medium
FBS: Fetal bovine serum
DMSO: Dimethyl sulfoxide
PFA: Paraformaldehyde
NGS: Normal goat serum
DAPI: 4′,6-Diamidino-2-phenylindole
