# Supplementary material for: Effects of HDAC inhibitors on neuroblastoma SH-SY5Y cell differentiation into mature neurons via the Wnt signaling pathway
Source: BMC Neurosci. 2023 May 1;24:28. doi: 10.1186/s12868-023-00798-0 (PMC10152798; doi:10.1186/s12868-023-00798-0)
Supplement: Supplementary file 1 — Additional file 1. Additional figures. [file 12868_2023_798_MOESM1_ESM.pdf]

Additional file information

Effects of HDAC inhibitors on neuroblastoma SH-SY5Y cell differentiation into mature neurons via the Wnt signaling pathway

Jiyun Choi, Jinsu Hwang, Mahesh Ramalingam, Han-Seong Jeong[#], and Sujeong Jang*

Cocorrespondence: Han-Seong Jeong

Correspondence: Sujeong Jang

Email: sujeong.jjang@gmail.com

This Additional file information contains:

- **8 pages**
- **Additional file Figures (6 Figures, S1 to S6)**

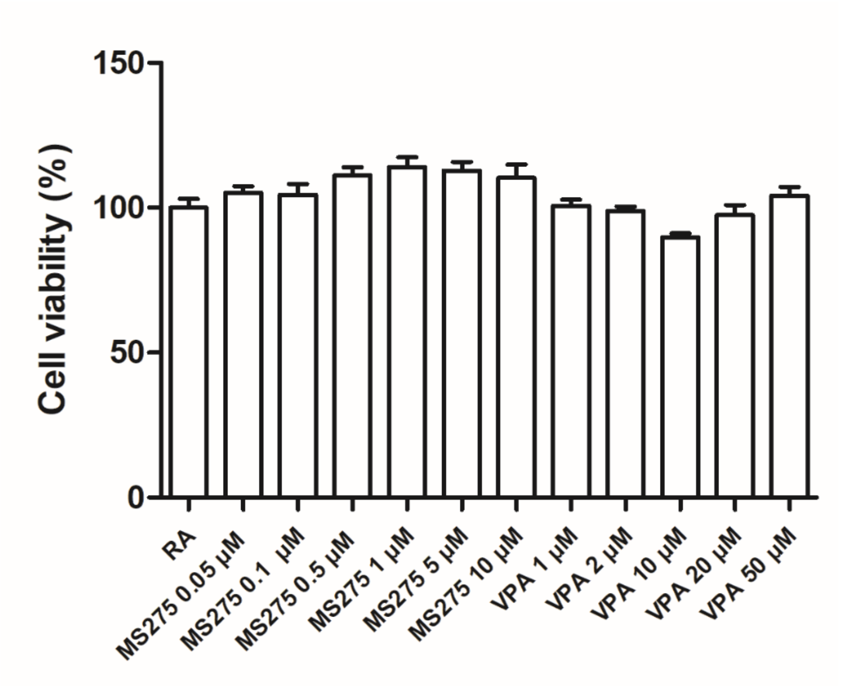

Fig. S1. The effect of HDAC inhibitors on cell viability. The number of cells was not decreased with different concentrations of HDAC inhibitors. There are no significant changes.

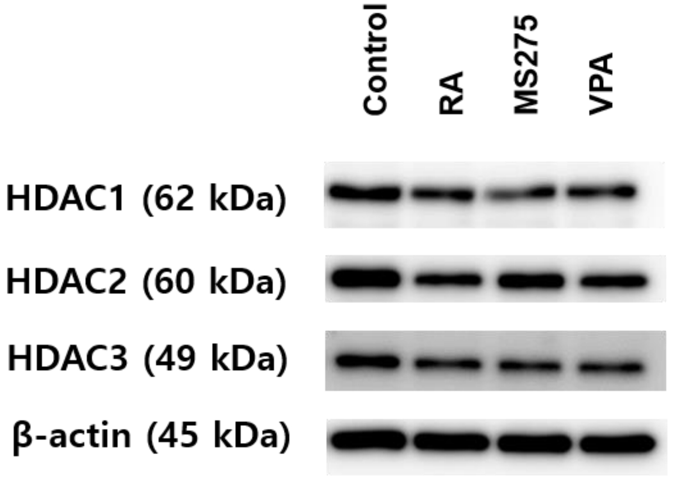

Fig. S2. The effect of HDAC inhibitors on histone modification. The treatment of HDAC inhibitors was increased HAT activation and decreased HDAC expression.

For Fig S2

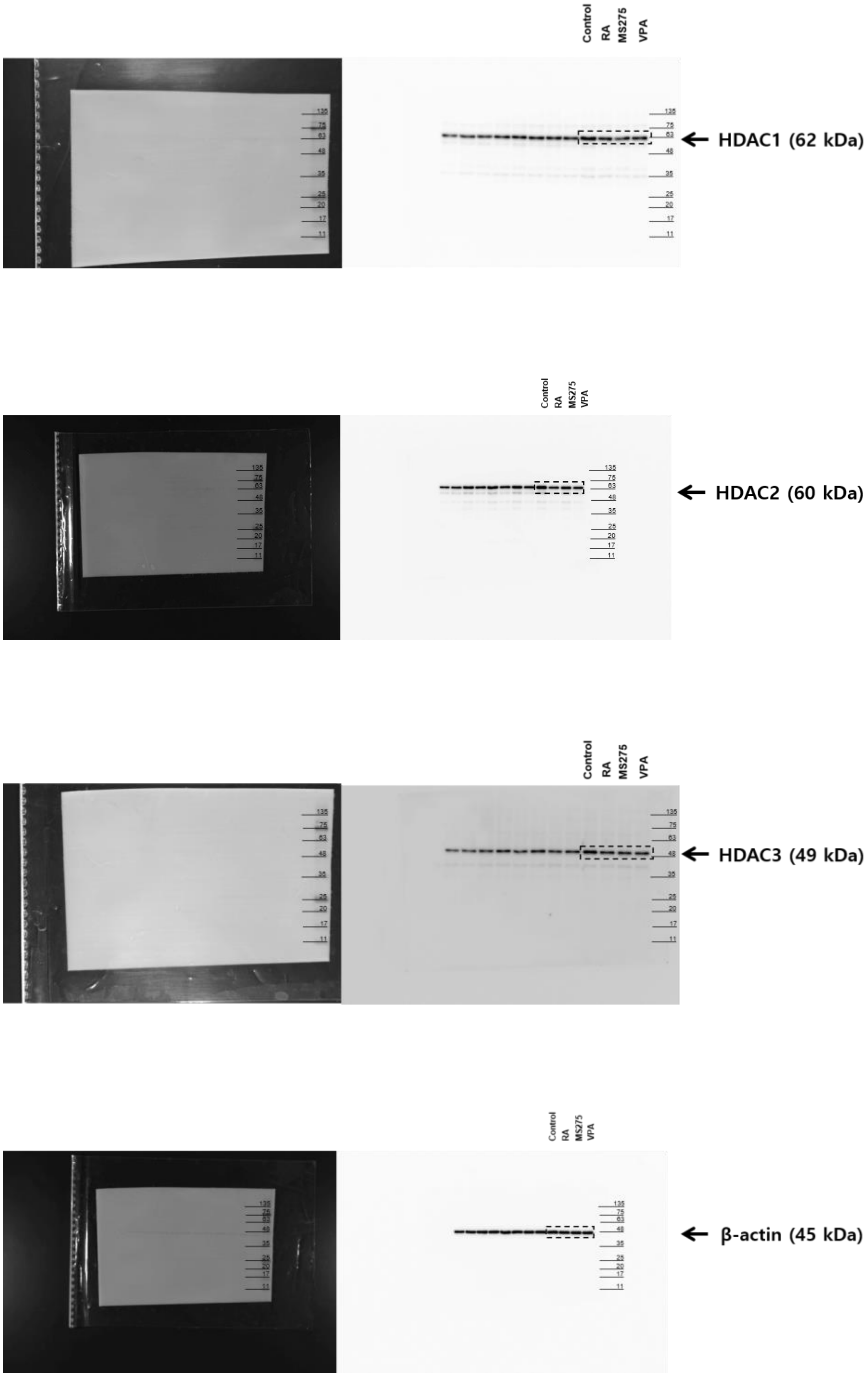

Fig. S3. Unedited images and their molecular markers for respective Western blots used in **Fig. S2** of this manuscript.

For Fig. 4a

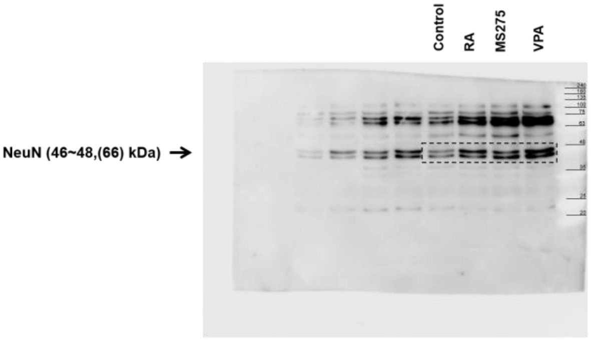

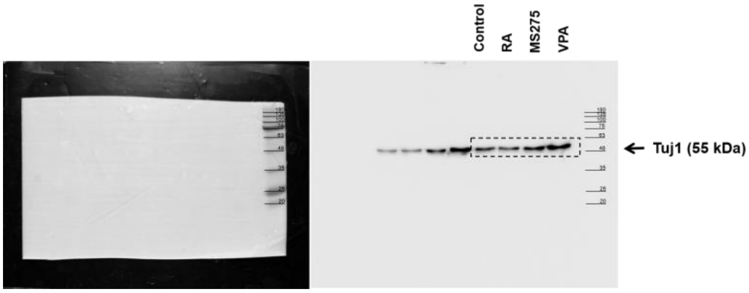

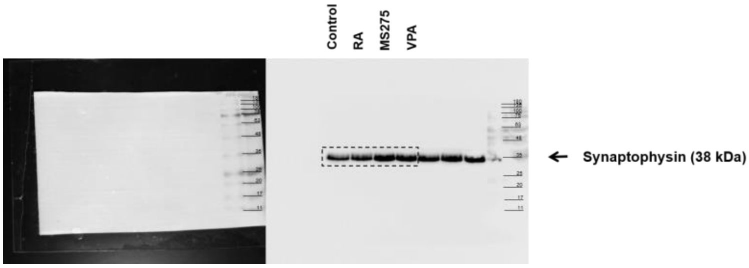

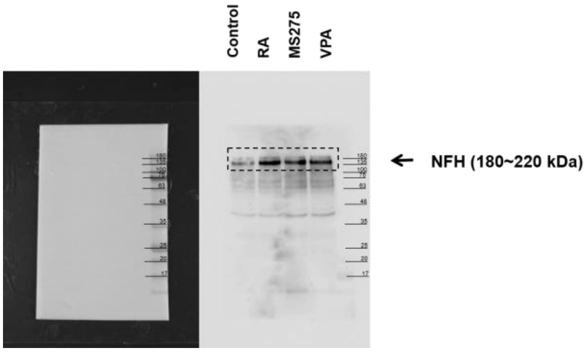

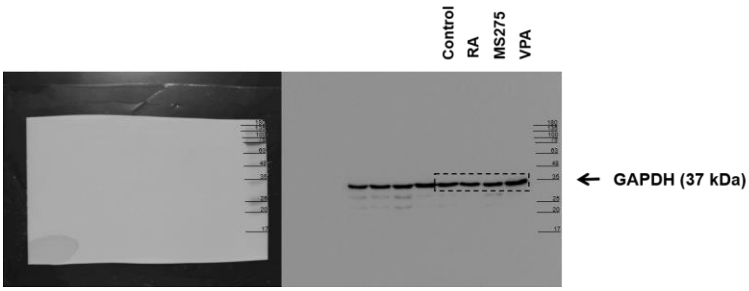

Fig. S4. Unedited images and their molecular markers for respective Western blots used in **Fig. 4a** of this manuscript.

For Fig. 4c

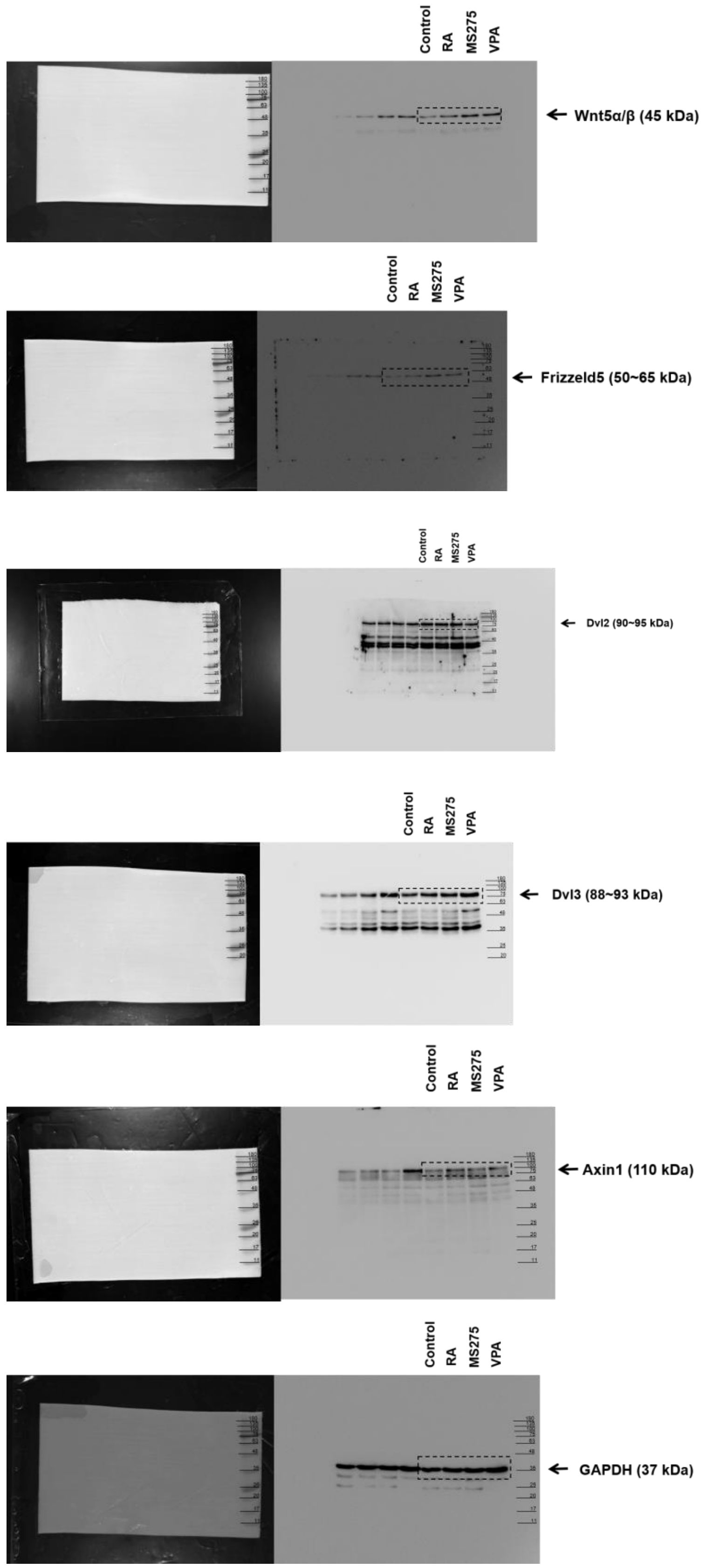

Fig. S5. Unedited images and their molecular markers for respective Western blots used in **Fig. 4c** of this manuscript.

For Fig. 4e

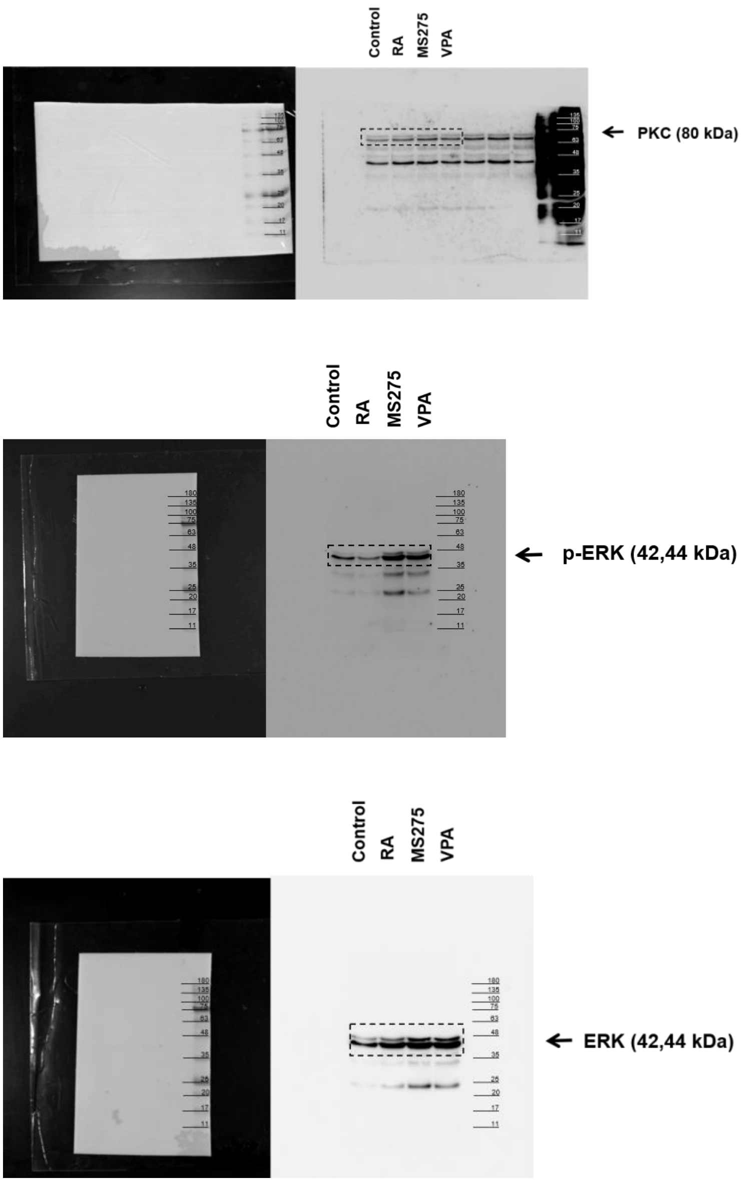

Fig. S6. Unedited images and their molecular markers for respective Western blots used in **Fig. 4e** of this manuscript.

For Fig. 4e

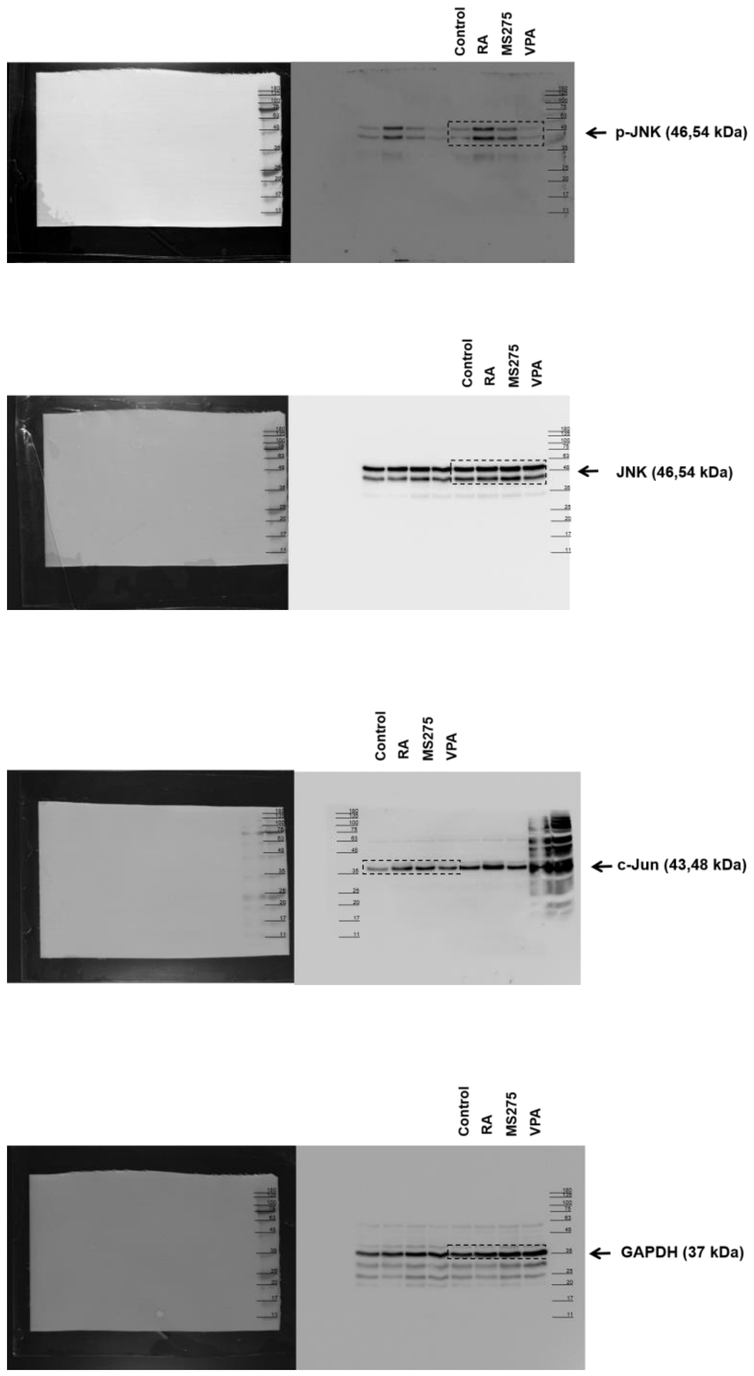

Fig. S6 (Continue). Unedited images and their molecular markers for respective Western blots used in **Fig. 4e** of this manuscript.
